# Customized BioRoot Inlay for the Management of an Immature Tooth With a Blunderbuss Canal: A Novel Biomimetic Approach

**DOI:** 10.7759/cureus.116158

**Published:** 2026-09-12

**Authors:** Sanjeev Srivastava, Shubhangi Shubhangi, Chirantan Chowdhury, Prachi Jha, Ranjeeta Aggarwal

**Affiliations:** 1 Department of Conservative Dentistry and Endodontics, Sardar Patel Post Graduate Institute of Dental and Medical Sciences, Lucknow, IND; 2 Department of Biostatistics, Coronation Institute of Innovative Learning, Lucknow, IND

**Keywords:** apexification, biodentine, bioroot inlay, blunderbuss canal, open apex

## Abstract

Management of immature teeth with open apices and severely divergent blunderbuss canals presents challenges in achieving predictable obturation and apical sealing. This case report describes a customized BioRoot inlay technique for the management of a previously treated immature maxillary central incisor with complex root canal anatomy. A 52-year-old female presented with discoloration of the maxillary left central incisor. The tooth had sustained traumatic injury during adolescence, resulting in pulpal necrosis and arrested root development with an incompletely formed apex. Root canal treatment was performed approximately 10 years before the present evaluation. Clinical and radiographic examination revealed inadequate previous obturation, a wide blunderbuss canal, external inflammatory root resorption, and a large periapical radiolucency. We performed nonsurgical retreatment, followed by chemomechanical debridement, intracanal calcium hydroxide medication, and local application of zoledronic acid. An intracanal impression was subsequently obtained using light-body polyvinyl siloxane. A customized BioRoot inlay was fabricated extraorally by condensing Biodentine into the impression-derived mold and was subsequently cemented within the canal. Postoperative periapical radiographic evaluation demonstrated satisfactory adaptation of the customized inlay and a satisfactory radiographic appearance of the apical obturation. The patient was followed clinically and radiographically, with favorable findings observed during follow-up. This customized BioRoot inlay technique provided a patient-specific method for managing the complex anatomy of an immature tooth with a blunderbuss canal. The technique may facilitate controlled placement and adaptation of the biomaterial within irregular canal anatomy. Further clinical studies with longer follow-up are required to determine its reproducibility and long-term clinical effectiveness.

## Introduction

An effective apical seal is fundamental to successful endodontic treatment; however, achieving predictable sealing in teeth with open apices remains clinically challenging. Pulpal necrosis before completion of root development, commonly resulting from trauma, deep caries, or developmental disturbances, arrests root maturation and may result in thin radicular dentin and a widely divergent apical foramen. The absence of a physiologic apical constriction complicates working-length determination, chemomechanical debridement, controlled placement of intracanal materials, and predictable obturation. These difficulties are especially pronounced in blunderbuss canals, characterized by a markedly flared, funnel-shaped morphology [1,2].

The primary objectives in managing permanent teeth with open apices are eliminating intracanal infection, preserving the remaining radicular structure, and establishing an effective apical seal. Apexification has traditionally been used for the treatment of necrotic immature permanent teeth. Conventional calcium hydroxide apexification can require prolonged treatment and multiple appointments and may increase the risk of structural weakening of already compromised roots [2]. Regenerative endodontic procedures (REPs) have emerged as a biologically based treatment option for selected immature permanent teeth with necrotic pulps and open apices, with the potential to promote continued root development and maturation. However, their applicability depends on appropriate case selection, adequate disinfection, and the presence of a suitable biological environment for tissue regeneration. The development of calcium silicate-based biomaterials, including mineral trioxide aggregate and other bioceramic materials, has facilitated more efficient creation of artificial apical barriers and has simplified the management of open apices [3].

Patient-specific approaches that conform to the existing canal morphology may therefore provide an alternative strategy for managing complex open apices. The BioRoot inlay technique involves the fabrication of a customized calcium silicate-based construct using an intracanal impression, with the aim of achieving individualized adaptation to the three-dimensional canal anatomy and facilitating controlled placement within a severely divergent canal. This case report describes the clinical application of a customized BioRoot inlay for the management of a previously treated immature maxillary central incisor with a blunderbuss canal, external inflammatory root resorption, and a large periapical lesion.

## Case presentation

A 52-year-old woman presented to the Department of Conservative Dentistry and Endodontics with a chief complaint of discoloration of the maxillary left central incisor (#21). The patient reported a history of traumatic injury to the maxillary left central incisor, followed by root canal treatment approximately 10 years before the present evaluation. The exact timing of the original traumatic injury and the specific details of the initial endodontic treatment were not available in the patient's records. At presentation, the tooth exhibited an incompletely developed root with a wide, blunderbuss canal, consistent with arrested root development following loss of pulp vitality before completion of root maturation. Clinical examination revealed discoloration of tooth #21, with no tenderness to percussion or palpation. No sinus tract or localized swelling was observed, and the tooth exhibited no clinically detectable mobility. The adjacent teeth demonstrated normal responses to pulp sensibility testing, with no associated clinical signs or symptoms (Figure 1A). Radiographic examination demonstrated inadequate previous obturation, a large periapical radiolucency, and an immature root with a wide blunderbuss canal and thin radicular dentinal walls. An irregular loss of external root structure was also evident, consistent with external inflammatory root resorption (Figure 1B). A diagnosis of a previously treated tooth with apical pathosis and an open apex was established, and nonsurgical endodontic retreatment using a customized BioRoot inlay was planned.

Written informed consent was obtained from the patient for the treatment procedure and for publication of the clinical details, radiographic findings, and clinical images associated with this case report.

Following rubber dam isolation, the existing gutta-percha was removed (Figure 1C). Working length was established, and minimal canal instrumentation was performed to preserve the remaining radicular dentin. Chemomechanical debridement was subsequently performed using 1% sodium hypochlorite followed by 17% ethylenediaminetetraacetic acid (EDTA). Calcium hydroxide was placed as an intracanal medicament, and the access cavity was temporarily sealed.

After two weeks, the patient remained asymptomatic. The intracanal medicament was removed, and the canal was thoroughly irrigated and dried. Considering the large periapical lesion with associated external inflammatory root resorption, 10⁻⁶ M zoledronic acid prepared in sterile saline was applied intracanal for three minutes (Figure 1D). As intracanal zoledronic acid is not an established endodontic treatment, it was used as a case-specific adjunct as an antiresorptive agent. Care was taken to minimize extrusion through the open apex, and the solution was subsequently removed by thorough irrigation before further treatment. The canal was subsequently irrigated with sterile saline and dried using absorbent paper points.

An intracanal impression was obtained using Aquasil Ultra XLV light-body polyvinyl siloxane (Dentsply Sirona Inc., York, Pennsylvania) delivered through an automixing tip (Figure 1E). A No. 40 H-file was used as a carrier to support the impression material during polymerization. The material was allowed to set for approximately five minutes according to the manufacturer's recommended setting characteristics. The impression was then carefully retrieved and inspected for integrity.

The obtained impression was embedded in addition silicone putty to create a stable mold. The light-body impression material and impression carrier were subsequently removed from the putty, leaving a negative mold reproducing the three-dimensional canal anatomy (Figure 1F). A customized BioRoot inlay was fabricated extraorally by incrementally condensing Biodentine (Septodont, Saint-Maur-des-Fossés, France) into the prepared mold. The material was allowed to set for approximately 12 minutes, corresponding to the manufacturer's stated setting time, before removal from the mold (Figure 1G). The fabricated inlay was carefully retrieved and trial-fitted to confirm passive adaptation within the canal (Figure 1H).

Before reinsertion, the customized inlay was disinfected with 2% chlorhexidine for 60 seconds and rinsed with sterile saline, after which Biodentine was mixed according to the manufacturer's instructions and applied as a cementing medium to the canal wall. The inlay was gently seated to its predetermined position without excessive apical pressure and was allowed to set completely. A postoperative periapical radiograph demonstrated satisfactory positioning and adaptation of the customized BioRoot inlay within the canal (Figure 1I). 

**Figure 1:**
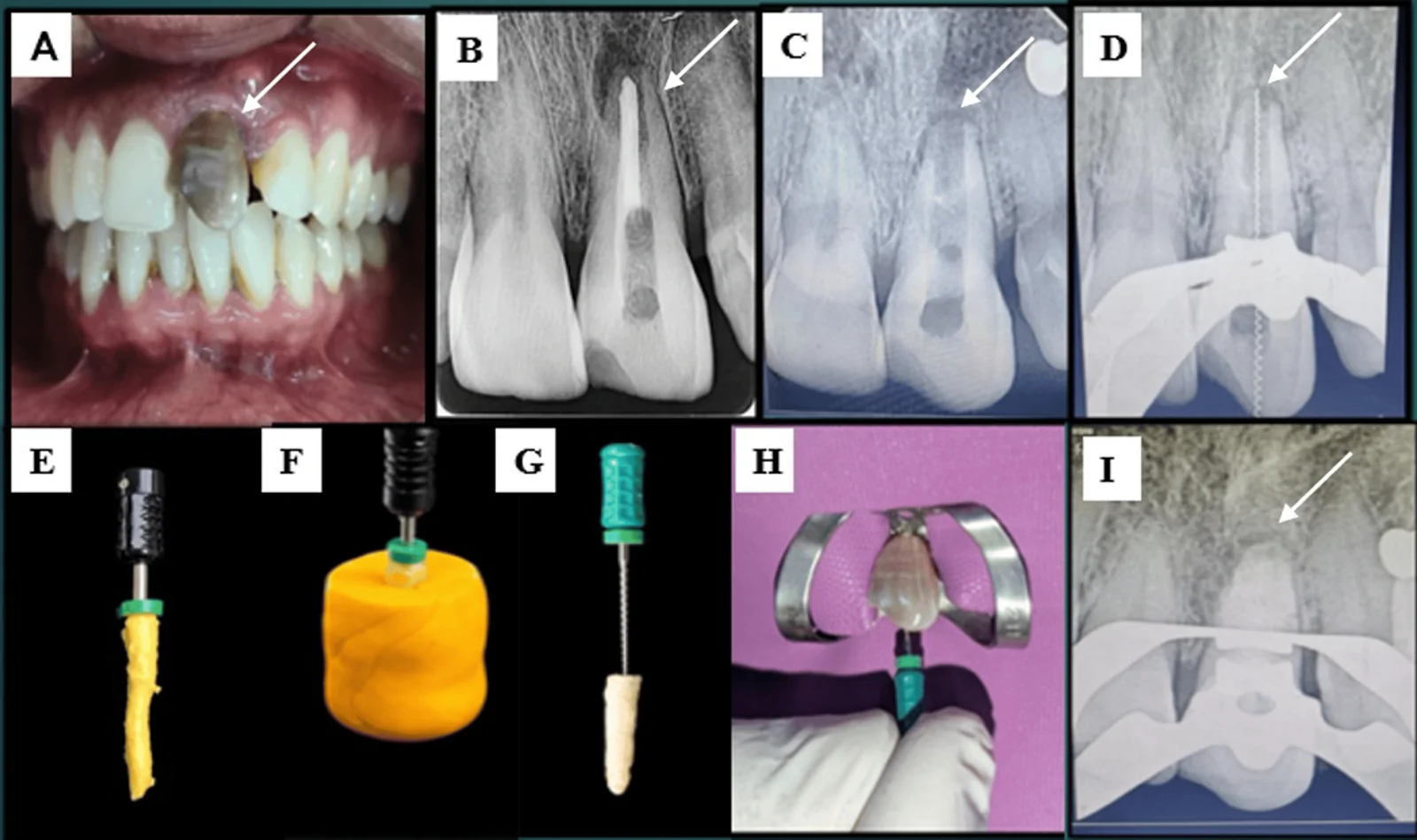
Clinical and radiographic sequence of the customized BioRoot inlay procedure (A) Preoperative clinical photograph showing discoloration of the maxillary left central incisor; (B) preoperative periapical radiograph showing inadequate previous obturation, a wide blunderbuss canal, open apex, and periapical radiolucency; (C) retrieval of the previous gutta-percha obturation; (D) intracanal application of zoledronic acid; (E) intracanal impression obtained using light-body polyvinyl siloxane; (F) fabrication of the customized BioRoot inlay using the impression-derived mold; (G) customized BioRoot inlay after fabrication; (H) clinical placement of the BioRoot inlay under rubber dam isolation; (I) postoperative periapical radiograph demonstrating the positioned BioRoot inlay. The arrow indicates the treated tooth.

The access cavity was restored with composite resin. At the two-month follow-up, the tooth remained clinically asymptomatic, with satisfactory radiographic findings (Figure 2A). At the four-month follow-up, the tooth remained asymptomatic, with continued satisfactory adaptation and reduction of the periapical radiolucency, indicating favorable periapical healing (Figure 2B). At the 12-month follow-up, the tooth remained clinically asymptomatic, and the definitive prosthetic restoration was maintained (Figure 2C). A standardized periapical radiograph demonstrated a reduction in the extent of the periapical radiolucency compared with the baseline radiograph, with no evident radiographic progression of the external inflammatory root resorption (Figure 2D). These findings were interpreted as radiographic evidence of periapical healing; however, complete resolution of the lesion or definitive arrest of the resorptive process was not established. 

**Figure 2:**
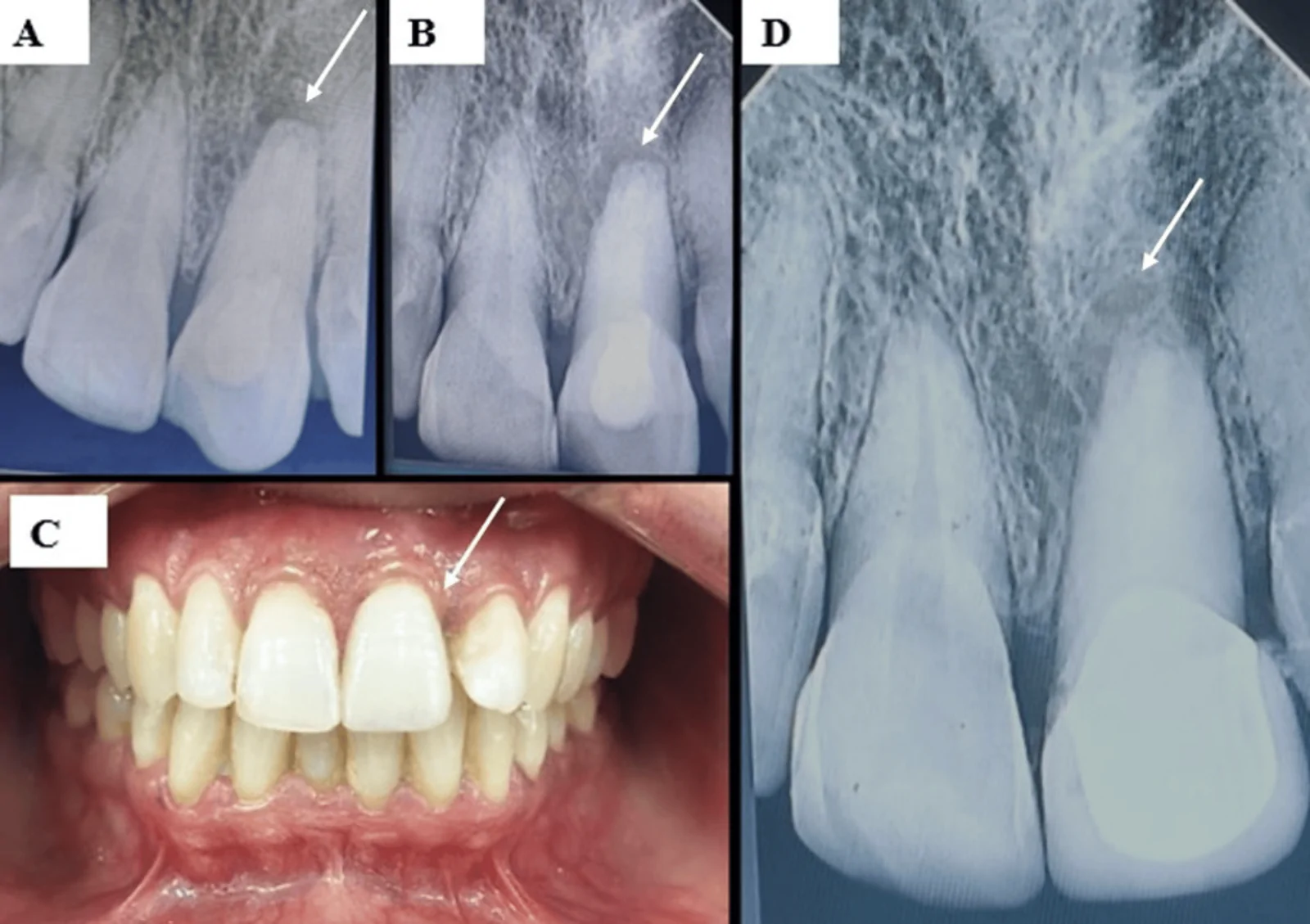
Clinical and radiographic follow-up after BioRoot inlay placement (A) Periapical radiograph at a two-month follow-up demonstrating the treated tooth; (B) periapical radiograph at a four-month follow-up demonstrating the treated tooth; (C) clinical photograph showing the definitive prosthetic restoration at the 12-month follow-up; (D) standardized periapical radiograph at the 12-month follow-up demonstrating reduction of the periapical radiolucency compared with baseline and no evident progression of the external inflammatory root resorption. The arrow indicates the treated tooth.

## Discussion

The present case demonstrates the clinical feasibility of a customized BioRoot inlay as an alternative approach for the management of a previously treated permanent tooth with a wide blunderbuss canal. The customized design enabled accurate adaptation to the complex canal morphology, overcoming one of the principal technical limitations associated with conventional orthograde placement of calcium silicate-based biomaterials in markedly flared canals.

Unlike one-step apexification, in which the biomaterial is incrementally condensed to create an apical barrier, the BioRoot inlay is fabricated extraorally from an intracanal impression, allowing controlled condensation, complete maturation, and facilitated verification before placement. This customized approach minimizes operator variability, reduces the risk of internal voids and distortion, and permits controlled insertion with minimal apical pressure, thereby limiting material extrusion beyond the open apex. The precise adaptation of the inlay to the canal walls maximizes contact with the remaining radicular dentin while minimizing interfacial gaps, resulting in improved sealing and a more uniform distribution of functional stresses. In addition, freshly mixed Biodentine serves as a luting agent, promoting biomineralization at the dentin-material interface to form a mineral infiltration zone that creates a monoblock-like restorative complex. This enhances stress transfer across the tooth-restoration interface, reduces microleakage, and may improve the fracture resistance and long-term prognosis of structurally compromised immature teeth [4-6].

The favorable outcome observed in the present case can also be attributed to the biological and physicochemical properties of Biodentine. Its rapid setting time, dimensional stability, and favorable handling characteristics make it well suited for fabrication of customized intracanal constructs [6]. Biodentine releases calcium ions during hydration, promoting hydroxyapatite deposition and formation of a mineral infiltration zone at the dentin-material interface, thereby enhancing interfacial integrity and sealing ability [5,6]. Furthermore, Biodentine stimulates the release of transforming growth factor-β1 (TGF-β1), promotes odontoblast-like cell differentiation, and upregulates mineralization-associated proteins, facilitating hard tissue deposition, apical mineralized barrier formation, and thus periapical healing [7]. Its dentin-like elastic modulus, low solubility, and sustained alkaline pH further contribute to favorable biomechanical performance and long-term structural stability [6].

An additional consideration in the management of immature teeth with extensive periapical pathology is the prevention of progressive inflammatory root resorption. Zoledronic acid, a third-generation nitrogen-containing bisphosphonate, has emerged as a potential adjunct because of its potent antiresorptive properties. It exerts its effect by inhibiting farnesyl pyrophosphate synthase within the mevalonate pathway, thereby preventing prenylation of small guanosine triphosphate (GTP)-binding proteins essential for osteoclast function. This results in osteoclast dysfunction, loss of the ruffled border, reduced bone resorption, and induction of osteoclast apoptosis [8]. Experimental studies have also demonstrated anti-inflammatory effects through suppression of pro-inflammatory cytokines and matrix metalloproteinases, suggesting a potential role in limiting inflammatory root resorption and promoting periapical healing. Although clinical evidence in endodontics remains limited, local application of zoledronic acid may represent a promising adjunct in selected cases exhibiting active external inflammatory resorption [8,9].

Although the clinical and radiographic findings of the present case are encouraging, the BioRoot inlay remains an emerging treatment concept. Further laboratory investigations evaluating interfacial adaptation, sealing ability, fracture resistance, and fatigue behavior, together with well-designed prospective clinical studies and long-term follow-up, are required to establish standardized clinical protocols and validate its long-term effectiveness.

## Conclusions

This case demonstrates the clinical feasibility of a customized BioRoot inlay for managing an immature tooth with a wide blunderbuss canal and open apex. The patient-specific fabrication approach facilitated adaptation of the bioceramic construct to the complex canal anatomy and allowed controlled placement within the canal. Although favorable clinical and radiographic findings were observed during follow-up, the technique should be considered an emerging treatment concept rather than an established alternative to conventional apexification. Further experimental studies and long-term clinical investigations are required to evaluate its sealing ability, mechanical performance, reproducibility, and long-term clinical outcomes before widespread clinical adoption.
